# S. C. Medical Society

**Published:** 1879-02-20

**Authors:** Francis L. Parker, J. L. Ancrum, W. H. Bailey, H. W. DeSaussure, R. A. Kinloch

**Affiliations:** President; Charleston, S. C.; Vice-President; Charleston, S. C.; Secretary; Charleston, S. C.; Treasurer; Charleston, S. C.; Charleston, S. C.


### *MEDICAL SOCIETY OF SOUTH CAROLINA.*

[CIRCULAR.]

*Charleston, S. C., December*, 4, 1878.

The undersigned, a Committee of the Medical Society of South Carolina, beg leave respectfully to submit to your consideration the accompanying Resolutions, passed at the regular meeting of the Society, on October 1st, 1878, and solicit contributions for the object therein stated.

The cause is one which appeals not to members of the Medical Profession alone, but to all charitably disposed persons, who admire, and are willing to reward self-sacrificing devotion and heroism in aid of sick and suffering humanity.

FRANCIS L. PARKER, M.D., *President.*
J. L. ANCRUM, M. D., *Vice-President.*
W. H. BAILEY, M.D., *Secretary.*
H. W. DESAUSSURE, M.D., JR., *Treasurer*
R. A. KINLOCH, M.D.

*Resolved*, 1. That the action of the Medical Society of the County of New York in raising a fund for the aid of the families of Physicians who have fallen, or may fall, victims to the epidemic of Yellow Fever meets with our warmest approval, and calls for our earliest co-operation.

*Resolved*, 2. That a Committee be appointed, composed of the Officers of this Society, to solicit contributions from our members, and the members of our Profession generally, or from any other persons who may be disposed to aid in this noble charity.

*Resolved*, 3. That the funds so raised, as also a copy of these Resolutions, be forwarded, as soon as practicable, to the President of the New York County Society, Dr. J. C. PETERS.

☞ Please subscribe, induce your friends to do so, and remit per express or P. O. money order.

Funds may be forwarded to Dr. H. A. DESAUSSURE, of Charleston, S. C., or directly to Dr. J. C. PETERS, President of the Medical Society of the County of New York, 83 Madison Avenue, which Society has taken noble action in this good work.

